# Variable humic product effects on maize structural biochemistry across annual weather patterns and soil types in two Iowa (U.S.A.) production fields

**DOI:** 10.3389/fpls.2022.1058141

**Published:** 2023-01-12

**Authors:** D. C. Olk, D. L. Dinnes, R. D. Hatfield, J. R. Scoresby, J. W. Darlington

**Affiliations:** ^1^ U.S. Department of Agriculture – Agricultural Research Service, National Laboratory for Agriculture and the Environment, Ames, IA, United States; ^2^ U.S. Department of Agriculture – Agricultural Research Service, U.S. Dairy Forage Research Center, Madison, WI, United States; ^3^ Retired, Princeton, WI, United States; ^4^ Minerals Technologies, Inc., New York, NY, United States; ^5^ Retired, Homedale, ID, United States

**Keywords:** humic product, maize, phenols, carbohydrates, lignification

## Abstract

Agronomic benefits of humic product application to crops are receiving increasing attention, though underlying biochemical changes remain unexplored, especially in field settings. In this study, maize (Zea mays L.) concentrations of 11 phenol and five carbohydrate monomers were determined in whole plant stover (four growing seasons) and roots (two growing seasons) at physiological maturity for two rainfed fields in Iowa (USA) having humic product applications. Stover and root tissues tended toward greater phenol concentrations in a drier upland transect but greater carbohydrate concentrations in a wetter lowland transect. Two humic treatments further accentuated these trends in upland roots. Their phenol content increased significantly with humic application in the droughtier season of root sampling (2013). Phenol increases above the unamended control averaged 20% for each monomer. Total phenols increased above the control by 12% and 19% for the two humic treatments. Five carbohydrate monomers in the upland roots did not respond to humic application. In the second year of root sampling (2014), which had abundant rainfall, upland root phenols did not respond substantively to humic application, but root carbohydrates increased on average by 11 or 20% for the two humic treatments compared to the control, reaching significance (P< 0.10) in 7 of 10 cases. Upland stover phenol concentrations responded differently to humic product application in each of four years, ranging from numeric increases in the droughtiest year (2012) to significant decreases with abundant rainfall (2014). In the lowland transect, root phenols and carbohydrates and stover phenols responded inconsistently to humic application in four years. Stover carbohydrates did not respond consistently to humic application in either transect. The phenols that were more responsive to humic application or to droughtier conditions included p-coumaric acid and syringaldehyde, which are heavily involved in late-season maize lignification. In summary, humic product application further promoted root lignification, a natural response to drought. Yet under non-drought conditions it promoted root carbohydrate production. Carbohydrate production might be the intrinsic plant response to humic product application in stress-free conditions. These results indicate complex interactions in field conditions between plant biochemistry, environmental signals, and the humic product.

## Introduction

Humic products have received increasing attention as potential field amendments for increasing crop growth and economic yield. Their efficacy in promoting plant growth has been demonstrated most commonly under controlled conditions (Chen and Aviad, 1990; Rose et al., 2014). A modest yet increasing number of field studies have also demonstrated positive crop responses for horticultural (Canellas et al., 2015) and other agronomic and pasture crops (Calvo et al., 2014; Olk et al., 2018). In one of the few multi-year field studies, near Ames, Iowa, in the U.S. Corn Belt our previous work (Olk et al., 2021) found that the benefit of one humic product to maize (*Zea mays* (L.) growth and grain yield on highly productive soils was best expressed in droughtier years and landscape positions. In our second agronomic study, Olk et al. (2022) reported that maize grain yields, as measured through yield components in production fields, increased modestly in central Iowa in response to a second humic product in each of three years.

**Figure 1:**
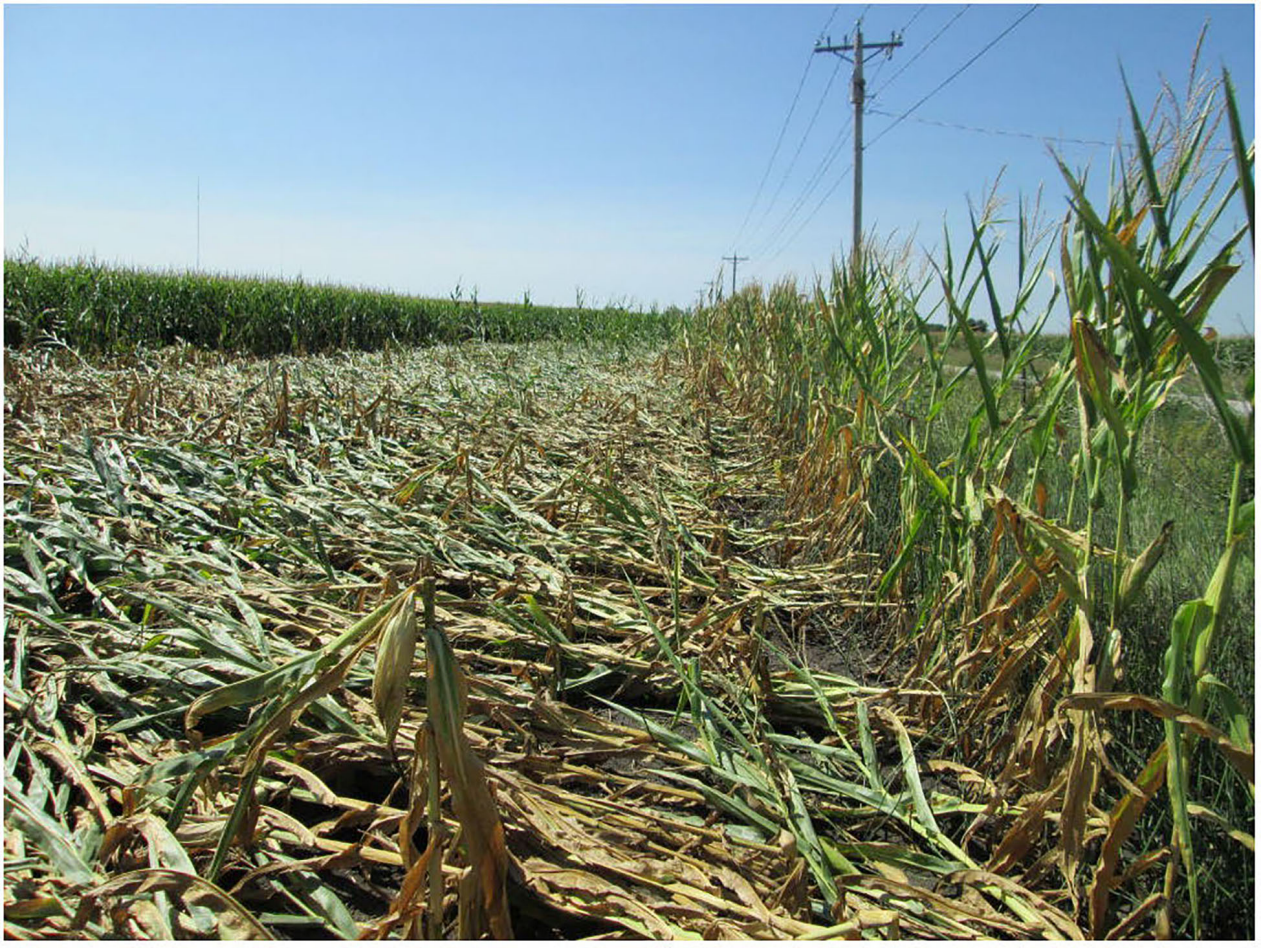
Wind damage to 16 border maize rows lacking humic product application in an upland area of a production field at a reproductive growth stage, 2012. The wind direction was from right to left. The first standing maize row (left) was the first row that had received the humic product.

During the first year (2012) of the study by Olk et al. (2021), a strong windstorm damaged maize growth at a reproductive growth stage. On a side slope position, the storm knocked down unamended maize plants in all 16 border rows on the field edge facing the storm (**Figure 1**). The intensity and duration of the wind were sufficiently strong that the stalks were irreversibly bent (but not severed) only 10-20 cm above the soil surface. The first maize row that withstood the wind was the 17th row into the field, which was the first row that had received the humic product (**Figure 1**). Yet in a lowland position at the base of that slope, maize plants were felled across much of the research plots, including both treated plots and unamended controls. One possible interpretation is that humic product application promoted lignification of the maize plants in the droughtier upland location. This hypothesis prompted further study of structural biochemical responses of maize to the humic product across the four years of this field trial.

Humic substance effects on plant physiology are well known, as reviewed by Calvo et al. (2014) and Nardi et al. (2016). Physiological effects include increased photosynthesis, heightened activities of ATPases and several other enzymes, altered production rates of antioxidants, and selective gene activation. Specifically for plant biochemistry, in controlled conditions, Schiavon et al. (2010) and Ertani et al. (2011) found that addition of humic materials derived from earthworm feces or lignosulphonates, respectively, to young maize plants caused short-term increases in phenolic compounds and carbohydrates. Both using humic products of unstated origin, Sakr et al. (2021) reported that a humic product reversed deleterious effects of drought stress on broccoli (*Brassica oleracea* var. italica) physiology and biochemistry in a greenhouse study, including increased total phenols and soluble carbohydrates, while Irani et al. (2021) found the same for grapevines (*Vitis vinifera* L.) in a vineyard. However, Aminifard et al. (2012) found no effect of a humic product derived from leonardite on total phenols and total carbohydrates in hot pepper (*Capsicum annuum* L.) in field conditions. We are unaware of any other field studies of humic product effects on crop structural biochemistry.

To better understand the apparent increased wind resistance induced by humic product application in 2012, as described above, in this study, we focus on structural biochemical responses of maize to humic product application. Specifically, we analyzed maize yield component samples collected at physiological maturity in four growing seasons, as described by Olk et al. (2021), for their contents of lignin monomers as an index of plant lignification for corn stover. We also measured five carbohydrate monomers, as lignification is based on diversion of carbohydrates to produce phenols. In two of these four years, we also excavated maize roots in these field trials as part of a different study, and their lignin and carbohydrate responses to humic product application are also described in this study. These measurements were performed for an unamended control compared to the amendment of a previous formulation of the micronized humic product, Enersol^®^ ^1^, created through extremely fine grinding of leonardite ore. The measurements were conducted during four growing seasons in two production fields owned and managed by the same farm operator but in opposite phases of a maize− soybean *Glycine Max* (L.) Merr.) annual crop rotation in central Iowa. Both fields featured multiple soil types lying along elevational changes in spatial patterns that allowed all experimental treatments to traverse all soil types. Annual precipitation varied among the four years from severe drought to highly favorable. We hypothesized that humic product effects on maize structural biochemical composition would vary across annual weather patterns and soil types.

## Materials and methods

### Field sites

Details on the field locations, experimental designs, soil types, and management practices were reported by Olk et al. (2021). In summary, two on-farm sites were located in central Iowa (42° 02’ N, 93° 37’ W)–one slightly west of Ames, IA and another near Kelley, IA, that were separated by a distance of 5.5 km. Both fields are located on glacial till of the Des Moines Lobe (Wisconsin glaciation) and thus have similar geology, soils, and climate (Hatfield et al., 1999; Eidem et al., 1999).

**Figure 2:**
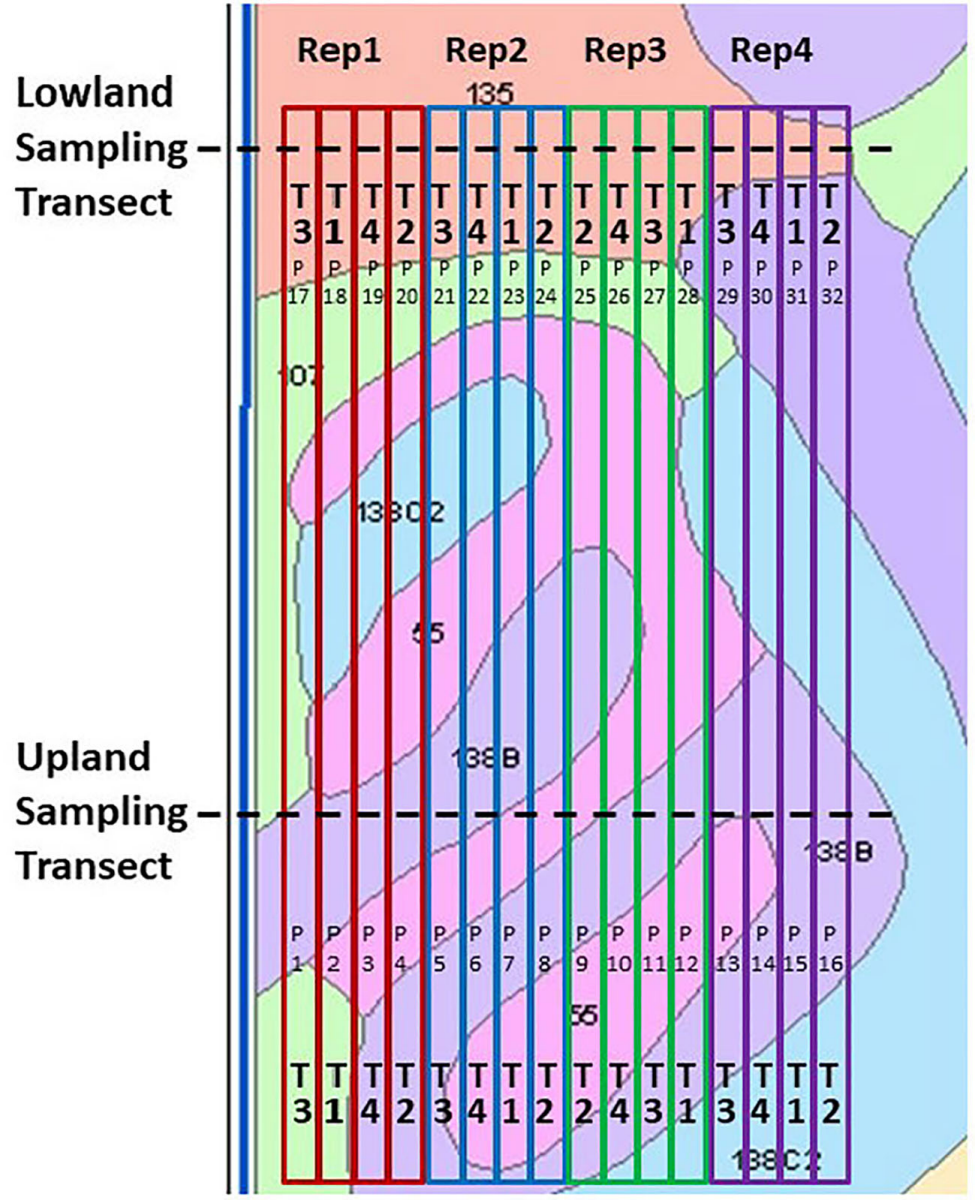
Field design of the Ames on-farm field research site, shown with soil mapping units and boundaries of the field-long treatment strips. Key to treatments (“T”): T1, Control lacking Enersol humic product application; T2, lower rate of Enersol humic product application at the fourth maize leaf stage; T3, split application (2012, 2014) or higher rate (2016) of humic product application; and T4, a separate alkali-extracted humic product. Plot numbers are shown as “P”. Key to soil mapping units: 55, Nicollet loam, 1 to 3 percent slopes; 107, Webster silty clay loam, 0 to 2 percent slopes; 135, Coland clay loam, 0 to 2 percent slopes, 138B, Clarion loam, 2 to 5 percent slopes; and 138C2, Clarion loam, 5 to 9 percent slopes, moderately eroded. Reprinted from Olk et al. (2021).

Both fields were in a maize−soybean crop rotation but in alternating years, and all analyses were conducted in the maize phase. Field-long treatment strips (380 m length x 6.1 m width) were located at the primary site near Ames to transect a continuum of the hilltop Clarion loam (fine-loamy, mixed, superactive, mesic Typic Hapludoll 2 to 5 percent slopes), side slope Nicollet loam (fine-loamy, mixed, superactive, mesic Aquic Hapludoll), and lowland Webster (silty clay loam fine-loamy, mixed, superactive, mesic Typic Endoaquoll) (**Figure 2**). The site near Kelley had a similar design, as the field-long treatment strips (343 m length x 6.1 m width) included both the hilltop Clarion loam (5 to 9 percent slopes, moderately eroded) and a lowland pattern of the Canisteo silty clay loam (fine-loamy, mixed, superactive, calcareous, mesic Typic Endoaquoll) and Harps loam (fine-loamy, mixed, superactive, mesic Typic Calciaquoll). The Kelley field did not include side slope soils. Clarion soils are well-drained. Nicollet soils are typically highly productive, due to favorable fertility and soil-water relations. Webster, Canisteo and Harps soils occur in low-lying, flat areas and are all poorly drained. The predominant textural classes in their uppermost 100 cm are loam for the Clarion, loam to clay loam for the Nicollet, silty clay loam and clay loam for the Webster, silty clay loam, clay loam, and loam for the Canisteo, and loam, clay loam, and sandy clay loam for the Harps (Soil Conservation Service, 1985), indicating increasingly finer soil textures downslope.

In both fields, the treatments were arranged in randomized complete block designs. Sampling transects for hand collection of plant and soil samples were established in two areas designated as either an upland or lowland landscape in each field, omitting the side slope Nicollet soil in the Ames field. Henceforth, each transect will be presumed as interchangeable with its respective soil type and landscape.

This study included the maize crop years of 2012, 2014 and 2016 for the Ames site and the 2013 maize crop year for the Kelley site. In 2015 the Kelley site was converted to continuous maize cropping and therefore was no longer included in this study. Precipitation patterns varied among the years, creating varying levels of perceived drought stress. The 2012 growing season was dominated by severe drought, the 2013 growing season was wet early followed by moderate drought, the 2014 growing season was favorable throughout except for seasonal wetness in a portion of the lowland transect, and the 2016 growing season had moderate drought early followed by favorable conditions throughout the remainder of the growing season.

Both experimental designs were imbedded within production fields operated by one commercial farming family, which followed its normal farming operations for the duration of this study, including conventional tillage. Maize row spacing was always at 0.76 m, and all field-long treatment strip-plots were eight rows wide.

The humic product used in this study was created through media milling of a naturally occurring leonardite ore from Gascoyne, North Dakota (United States). The native pH of the ore is 3.5–5.0; thus, the milling generates an acidic, aqueous suspension concentrate. It contains about 28% leonardite solid particles, which include at least 180 g kg^-1^ humic acid, at least 15 g kg^-1^ fulvic acid, 4 g kg^-1^ S, and 4 g kg^-1^ Ca. At the Ames field there were four replications of a control and three humic product treatments, whose application rates and timings were recommended by the manufacturer. Application timings are reported here following the leaf staging method that excludes the cotyledon leaf (Abendroth et al., 2011). The treatments in 2012 and 2014 were 2.5 L ha^-1^ Enersol humic product at the fourth maize leaf stage of vegetative growth (V4), 2 L ha^-1^ Enersol humic product at maize pre-emergence plus 1 L ha^-1^ Enersol humic product at V4 (“split”), 3 L ha^-1^ of a separate alkali-extracted humic product at V4, and an unamended control (**Figure 2**). Based on crop responses to Enersol at other sites, in 2016 the two application rates of the product were adjusted to 2.3 L ha^-1^ Enersol humic product at V4, 4.7 L ha^-1^ Enersol humic product at V4 (“high”), and 4.7 L ha^-1^ of a separate alkali-extracted humic product at V4. The alkali-extracted product treatment was more exploratory than were the other treatments, as the source of its extracted product varied among years. This treatment gave roughly analogous results as did the two Enersol treatments in the three years of the Ames field. Due to its variable sources, however, its results are not presented in this report.

In 2013 the Kelley field had four replications of three treatments that were also studied at the Ames site in 2012 and 2014, namely an untreated control, 2.5 L ha^-1^ Enersol humic product at V4, and 2 L ha^-1^ Enersol at maize pre-emergence plus 1 L ha^-1^ Enersol at V4 (split).

The locations of the field-long strip plots and sampling transects in both fields were marked by Global Positioning System and geographic information system technologies in each year to ensure the same locations of treatment strips across years.

### Fertilization rates and timing

In the autumns prior to the spring plantings of the maize crops, N-phosphorus (P)-potassium (K) fertilizers were applied following soybean harvest at the respective rates of 112-90-134 kg ha^-1^. An additional 67 kg N ha^-1^ was added in the spring just prior to maize planting in conjunction with pre-plant herbicide application, totaling 179 kg N ha^-1^ applied to the fields for maize production in the years 2012 through 2014. In 2016, approximately 4.9 Mg ha^-1^ of chicken (*Gallus gallus* domesticus) manure was applied to the Ames field on 4 April 2016, which was determined by later nutrient analyses to represent an effective N-P-K application rate of 28-67-56 kg ha^-1^. On 10 April 2016, an additional 134 kg N ha^-1^ of anhydrous ammonia ( $\text{N}{\text{H}}_{4}^{+}$ ) was injected into the field, thus totaling 162 kg N ha^-1^ applied to the field for the 2016 maize growing season. No additional P and K was applied in 2016 beyond that contained in the chicken manure.

### Plant sampling

Maize stover samples for this study were hand-harvested at physiological maturity each year for all treatment strips near both landscape sampling transects. In each treatment strip within each landscape transect, a one-row length of 1 m was harvested in areas of uniform growth by cutting seven evenly spaced plants at ground level. Whole-plant stover samples were oven-dried at 55°C under forced air, then immediately measured for oven-dry weights and mechanically shredded. Composite subsamples were taken of the shredded stover for later grinding through a Wiley mill (1 mm mesh screen, Thomas Scientific, Swedesboro, NJ, USA) and then a Cyclone mill (Udy Corporation, Fort Collins, CO, USA) to a powder consistency.

Root samples were collected in 2013 from the Kelley field and in 2014 from the Ames field as part of a separate study. In 2013, early season flooding damaged large sections of the lowland transect, allowing the relatively intensive root sampling only in the upland transect. In 2014, labor constraints allowed root sampling for the upland transect in the two Enersol application treatments and the unamended control, but for the lowland transect in only the split application treatment and the unamended control. In one maize row of representative crop growth for each field strip x landscape transect, a soil volume of 54 cm length, 30 cm width, and 30 cm depth was excavated to recover the roots from three adjacent maize plants at about the R2 maize growth stage. Root samples that were collected at two earlier crop growth stages will not be reported here. The soil blocks were transported from the field to a washing station, where the soil was washed through a 1-mm sieve to catch all living roots. The roots were transported indoors for fine washing, oven-drying, weighing, and fine grinding as with the stover samples.

### Plant biochemical analyses

Stover and root concentrations of carbohydrates were determined by anion chromatographic separation and pulsed amperometric detection, following the two-step weak-acid and strong-acid digestion of Martens and Loeffelmann (2002). Hemicelluloses (arabinose, galactose, xylose, and weak-acid glucose) were first extracted from stover or root samples by incubating 20-mg samples with 800 µL of 6 M H_2_SO_4_ for 30 min in test tubes at ambient temperature, then diluting with 4.0 mL deionized water to a final concentration of 1.0 M H_2_SO_4_ and autoclaving at 121°C for 30 min. Each digest was centrifuged and the supernatant transferred to a new test tube. The digested plant material was washed twice with 1.0 mL of deionized water. Following centrifuging, each supernatant was added to the first supernatant. The combined supernatant was neutralized to pH 5.5-6.5 using NaOH. The remaining plant residue was dried overnight at 60°C. To extract strong-acid glucose, which has been debated as an approximation of cellulose (Chantigny and Angers, 2008), the remaining plant residue was incubated with 300 µL of 18 M H_2_SO_4_ for 30 min, diluted with 3.3 mL deionized water to a final concentration of 1.5 M H_2_SO_4_ and autoclaved at 121°C for 30 min. Similar to the weak-acid extraction, the plant digests were centrifuged and washed to form a combined supernatant, then neutralized to pH 5.5-6.5. All extracts from both digestions were then filtered using a 0.2 µm syringe filter, and frozen until analysis by anion chromatography for concentrations of carbohydrate monomers, using a CarboPac PA10 column (2 mm i.d., 250 mm) and an isocratic mobile phase of 3 mM NaOH.

Lignin-derived phenols were measured by adapting the cupric oxide oxidation procedure of Olk et al. (2009). Samples of 6 mg finely ground stover or roots were oxidized with alkaline cupric oxide and 2 M NaOH for 3 h in a stainless steel mini-bomb. Bombs were loaded into a specially designed carousel in a refitted Hewlett-Packard 5890A gas chromatograph (GC). The heating rate was 4.2°C min^-1^ for 30 min from a start temp of 27°C, and a final temp of 150°C was held for 150 min. Following heating, 100 μl ethyl vanillin at a concentration of 240 ng μl^-1^ was added to each sample as the internal standard. The mini-bombs were closed again, shaken by hand, then centrifuged for 5 min at 3500 rpm, and their supernatant transferred to test tubes. Three ml of 6 M HCl was added to each bomb to neutralize the NaOH and each test tube was vortexed. About 2.5 g NaCl was added to each test tube, then capped and mixed by hand. About 3 ml ether was added, then again capped and mixed by hand. Test tubes were shaken for 2 minutes to accumulate the phenols in the ether, then centrifuged for 2 minutes at 2,000 rpm. The ether layer containing the phenols was then carefully pipetted from the surface of each solution into a new test tube. Na_2_SO_4_ (~2.5 g) was added to each tube to remove residual H_2_O from the ether solution. Tubes were shaken, and if necessary stored under refrigeration for overnight.

Samples were filtered through quartz wool and concentrated by rotary evaporation to about 1 ml volume, then transferred to 4-ml vials, and dried to nearly air-dryness under a stream of Ar gas. The precipitate was then dissolved in 300 μl pyridine/100 μl methyl-3,4-dimethoxybenzoate. The methyl-3,4-dimethoxybenzoate served as a recovery standard. A 50 μl aliquot of each solution was added to a new 4-ml vial and mixed with 50 μl of the derivatizing agent bis(trimethylsilyl)trifluoroacetamide (BSTFA) and then placed in a heating block at 70°C for 30 min, after which the samples were loaded into the GC. Gas chromatography was carried out with a Hewlett-Packard 6890 GC equipped with a derivatized, nonpacked injection liner, a HP-5 (5% crosslinked phenylmethyl siloxane) capillary column (30-m length, 0.32-mm column id., 0.25-μm film thickness) and a flame ionization detector. The following conditions were employed for phenol separation: injector temperature, 300°C; temperature ramp, 100°C ramped to 220°C at 4°C min^-1^, 220°C ramped to 270°C at 25°C min^-1^ and held for 10.1 min; detector temperature 300°C. The samples were analyzed for their concentrations of two cinnamic phenols (ferulic acid, p-coumaric acid), and aldehyde, ketone, and acid forms of syringyl, vanillyl, and p-hydroxybenzoic phenols. Results are reported in the conventional units of mg phenol 100 mg^-1^ plant organic C.

Lignin content and concentrations of ferulic acid and p-coumaric acid were also measured through alternative approaches at the milk reproductive (R3) growth stage in the 2013 upland transect. The first leaf above the primary ear (generally the 10^th^ or 11^th^ leaf for this maize variety), its sheath, and the underlying stalk section between that leaf and the ear (“internode”) were sampled from three representative plants in each plot. Each end of the internode was cut about 0.5 cm from the adjacent stalk node, and the overlying leaf sheath was separated. Samples were frozen, then freeze-dried and ground through a Cyclone mill to a fine powder.

Lignin contents of the internode and leaf samples were analyzed by the acetyl bromide method (Fukushima and Hatfield, 2004). Briefly, cell walls from leaf or internode samples were prepared by sequential extraction of the leaf or internode samples with water, ethanol, chloroform, and acetone in a Soxhlet apparatus. Then 100 mg of the cell wall preparation was digested with 4.0 mL of 25% acetyl bromide in glacial acetic acid at 50°C for 2 h, with occasional mixing. After cooling, the volume was made up to 16.0 mL volume with acetic acid and centrifuged (3000g, 15 min). Then 0.5 mL of this solution was added to a tube containing 2.5 mL of acetic acid and 1.5 mL of 0.3 M NaOH. After shaking, 0.5 mL of 0.5 M hydroxylamine hydrochloride solution was added, and the volume made up to 10 mL with acetic acid. Optical density at 280 nm was measured and concentration determined from the respective extinction coefficient that been calculated from standard curves.

Ferulic acid and p-coumaric acid were measured by NaOH extraction and gas chromatography through a modification of the method by Jung and Phillips (2010). Leaf, sheath, and internode subsamples were quantitatively added to 2-mL bead-beater microfuge tubes, further ground through bead beating, then amended with 1 mL 2 M NaOH spiked with mercaptolethanol (14 µL per 40 mL NaOH). Following mixing, the tubes were incubated for 20-24 h in a water bath (39°C), 600 µL 6 M HCl was added to lower the solution pH to less than 3.0, followed by gentle vortexing. The tubes were placed in a refrigerator for 2 h, then centrifuged for 10 min at 12,000 g. Supernatants were transferred to 1.7 mL centrifuge tubes, spiked with 25 µL of a 2-OH o-coumaric acid standard (1 mg mL^-1^), and centrifuged for 7 min at 12-15,000 g. The supernatants were passed through solid-phase extraction (ENVI-18 columns prepped with 1 mL 100% methanol and then 1 mL acidified water (pH< 2.0)) and rinsed once with 400 µL acidified water. Samples were dried and derivatized before analysis by GC.

### Statistical analyses

Treatments were randomized by individual treatment strip within each replication, but not re-randomized by each landscape transect. Therefore, the experimental designs are treatments nested within treatment strips, and the program for SAS Mixed Models statistical analyses (SAS Institute, 2012) was accordingly adjusted to the proper degrees of freedom for this design. Additional analyses were conducted by individual time or landscape transect to further examine treatment differences.

Several crop parameters showed consistent positive responses to the humic product that were not quite significant at P ≤ 0.10 but would have been significant at less stringent thresholds. Due to their consistency, we also discuss these numeric trends, including the separation of results by landscape transect, even in those cases having insignificant treatment−landscape interactions. In our previous work, the effects of humic products on crop growth were at times modest, but their magnitudes were modified gradationally by local environmental conditions in logical manners (Olk et al., 2021; Olk et al., 2022). This information on gradational responses will be useful for future comparisons of humic product efficacy in other field studies and would be lost by adhering strictly to pre-set levels of significance. Therefore, we will use the following categories to describe the significance of values: >0.2, nonsignificant; 0.2-0.1, near significant; 0.1-0.05, significant; 0.05-0.01, strongly significant; and <0.01, highly significant.

## Results

### Distinction of results by landscape

Results will be reported primarily by individual transect, as root sampling in the lowland transect was incomplete. Early season flooding in 2013 damaged the crop stand in the lowland landscape to the extent that the remaining crop stand sufficed only for collecting yield components (Olk et al., 2021). In 2014, mild flooding in the early season and labor constraints compelled omission of the 2.5 L ha^-1^ humic product treatment in the lowland transect. By contrast, root sampling in the upland transect was complete for both 2013 and 2014, the two years in which labor was available for root sampling. Hence, subsequent sections will report results separately for each of the transects, beginning with the more complete samplings in the upland transect.

Averaged across both humic product treatments and the unamended control, the landscape effect between the upland and lowland transects was frequently of statistical significance, which further encourages separate reporting of landscape transects. For the 11 stover phenols measured by CuO oxidation, the landscape effect was strongly significant (P<0.05) and often highly significant (P<0.01) for nine phenols in 2013, only three phenols in 2014, but it was highly significant (P<0.01) for 10 phenols in 2016 and also for total phenols (P11) in both 2013 and 2016 (**Table 1**). The phenol concentration was greater in the upland landscape than in the lowland landscape for 22 of these 25 significant cases. Trends were relatively uniform across all phenols. In 2012, the droughtiest year, stover phenol concentrations were clearly greater than for stover concentrations in all other years. This increase was especially strong in the lowland transect. Thus, none of the phenols differed significantly (P>0.10) by landscape in 2012, although their sum (P11) was significantly greater (P=0.041) in the lowland landscape than in the upland. For solely the control, stover concentrations of the 11 individual phenols were numerically greater in the upland transect than in the lowland transect for 31 of the 33 cases in 2013, 2014, and 2016. Of these, 21 cases reached significance (P<0.10), mostly in the droughtier years 2013 and 2016 (data not shown), and eight additional cases reached near significance (0.10<P<0.20), including five cases in 2014. For 2013, 2014, and 2016, total phenols in the control increased from the lowland to the upland by 22, 7, and 30%, respectively.

**Table 1:** Landscape means, averaged across the control and two humic product treatments, for concentrations of phenol compounds (mg phenol 100 mg^-1^ plant organic C) in maize stover, shown for four years for the upland (Up) and lowland (Low) transects, and the significance (P) of the landscape effect.

Phenol	2012			2013			2014			2016		
	Up	Low	P	Up	Low	P	Up	Low	P	Up	Low	P
Syringaldehyde	2.060	2.081	0.880	1,675	1.416	0.001	1.578	1.525	0.389	0.964	0.677	<0.0001
Acetosyringone	1.129	1.038	0.244	0.727	0.696	0.150	0.734	0.730	0.856	0.496	0.348	<0.0001
Syringic acid	0.677	0.729	0.510	0.498	0.425	0.002	0.470	0.441	0.099	0.294	0.179	<0.0001
Vanillin	1.481	1.433	0.809	1.233	1.144	0.040	1.190	1.161	0.446	0.920	0.676	<0.0001
Acetovanillone	0.184	0.186	0.972	0.160	0.145	0.003	0.149	0.152	0.317	0.121	0.090	<0.0001
Vanillic acid	0.228	0.232	0.912	0.160	0.145	0.004	0.160	0.161	0.099	0.111	0.073	<0.0001
Ferulic acid	1.229	1.250	0.963	1.156	1.031	0.002	0.938	1.035	0.007	0.718	0.539	<0.0001
p-Coumaric acid	3.256	3.659	0.223	2.652	2.200	0.001	2.405	2.491	0.303	1.622	1.389	0.001
p-OH-benzaldehyde	0.208	0.205	0.744	0.155	0.135	0.002	0.139	0.146	0.243	0.104	0.085	<0.0001
p-OH-acetophenone	0.023	0.031	0.499	0.026	0.023	0.015	0.023	0.026	0.536	0.015	0.017	0.002
p-OH-benzoic acid	0.066	0.067	0.999	0.032	0.031	0.156	0.034	0.032	0.461	0.027	0.016	<0.0001
Total (P11)	10.19	11.00	0.042	8.473	7.392	0.001	7.822	7.885	0.795	5.386	4.089	<0.0001

Color coding represents phenol families.

Similarly, in the sole year (2014) where root phenols were collected in both landscapes, the landscape effect across the control and both humic product treatments neared or reached significance (P<0.10) for three phenols having greater values in the upland transect, namely syringaldehyde (P=0.058), acetosyringone (P=0.052), and p-hydroxybenzaldehyde (P=0.107) (**Table 2**). Vanillic acid, however, was significantly greater (P=0.038) in the lowland transect. For the remaining seven phenols, the upland transect had numerically greater levels for five phenols than did the lowland transect. For solely the control, the greater values of the upland transect reached significance (P<0.10) in three cases and near significance (0.10<P<0.20) in two more of the 11 total cases.

**Table 2:** Landscape means, averaged across the control and two humic product treatments, for concentrations of phenol and carbohydrate compounds in maize roots for the 2014 growing season, for the upland (Up) and lowland (Low) transects, and the significance (P) of the landscape effect.

Compound	Up	Low	P
Phenols (mg phenol 100 mg^-1^ plant organic C)			
Syringaldehyde	2.372	2.221	0.058
Acetosyringone	0.660	0.616	0.052
Syringic acid	0.494	0.483	0.486
Vanillin	1.098	1.100	0.662
Acetovanillone	0.148	0.146	0.952
Vanillic acid	0.146	0.154	0.038
Ferulic acid	1.126	1.112	0.435
p-Coumaric acid	3.846	3.670	0.141
p-OH-benzaldehyde	0.238	0.228	0.107
p-OH-acetophenone	0.032	0.032	0.887
p-OH-benzoic acid	0.042	0.042	0.940
Total (P11)	10.203	9.834	0.199
Carbohydrates (g kg^-1^)			
Arabinose	17.9	18.9	0.097
Galactose	9.3	10.5	0.086
Xylose	92.6	119.3	0.012
Weak-acid glucose	54.9	41.3	0.164
Strong-acid glucose	159.0	190.3	0.012

Color coding represents phenol families.

The landscape effect across the control and both humic product treatments was also commonly significant among the carbohydrate results, however in the opposing direction from phenols: the upland transect had generally lower values. For the 2014 root carbohydrates, landscape effect was significant (P<0.10) for four of the five carbohydrate monomers, excepting weak-acid glucose (**Table 2**). For these four monomers, the lowland landscape had the greater values. For stover carbohydrates, the landscape effect neared or attained significance (P<0.10) for two of the five carbohydrate monomers in both 2012 and 2013, all five in 2014, and three of the five monomers in 2016 (**Table 3**). Of the four years, the landscape effect neared or reached significance for arabinose and weak-acid glucose in three years and for galactose, xylose and strong-acid glucose in two years. For each carbohydrate in each year, the lowland transect had numerically greater values than did the upland transect in 13 of 20 cases, although significance (P<0.10) was reached in only six cases. For only the control, the lowland transect had numerically greater carbohydrate concentrations in 12 of 20 cases for four years for stover, and four of five cases for the 2014 roots. Statistical significance (P<0.10) was reached in 11 stover cases and all four root cases. In general, stover and roots often tended toward greater phenols levels in the upland transect but greater carbohydrate levels in the lowland transect.

**Table 3:** Landscape means, averaged across the control and two humic product treatments, for concentrations of carbohydrate compounds (g kg^-1^) in maize stover, shown for four years for the upland (Up) and lowland (Low) transects, and the significance (P) of the landscape effect.

Carbohydrate	2012			2013			2014			2016		
	Up	Low	P	Up	Low	P	Up	Low	P	Up	Low	P
Arabinose	28.3	30.5	0.043	22.4	23.7	0.268	17.9	21.0	0.0003	19.3	17.8	0.106
Galactose	9.6	10.1	0.274	6.1	6.3	0.172	6.8	7.3	0.040	7.6	6.6	0.021
Xylose	143.7	166.4	0.152	94.7	100.4	0.051	103.5	120.4	0.004	89.0	85.8	0.600
Weak-acid glucose	128.0	137.8	0.790	39.2	54.8	0.021	43.8	25.8	0.0001	31.8	38.2	0.029
Strong-acid glucose	67.4	70.8	<0.0001	7.4	7.0	0.304	182.8	142.8	0.002	161.8	155.6	0.517

Despite the persistent significance of the landscape effect, we still report that the main plot treatment effect across both humic product applications and both landscapes was in most cases insignificant (P>0.10). For the 11 phenols in all four years, it was insignificant in 37 of 44 cases for the stover and all 11 cases for the 2014 roots. For the five carbohydrates in all four years, it was insignificant in 12 of the 20 cases, and it could not be calculated in four further cases, due in part to great numeric differences between the landscapes.

### Upland transect roots

#### Root phenol responses to the humic product

In the moderately droughty 2013 growing season, for 10 of the 11 phenol monomers and their sum (P11), the unamended control had the lowest concentrations (**Table 4**). The main plot effect with both humic product applications was significant (P<0.10) for the aldehyde and ketone forms of syringyl phenols and both cinnamic phenols. It neared significance (P<0.10) for the aldehyde and ketone forms of both the vanillyl and p-hydrobenzyl phenols. For each of the three families of phenols, the oxidized acid form (syringic acid, vanillic acid, p-hydroxybenzoic acid) had the worst level of significance. Paired t-tests with the control found significant (P<0.10) increases with the V4 application for 10 of the 11 phenols and their sum (P11), the sole exception being the near significance of vanillic acid (P=0.144). Again, the aldehyde and ketone forms of the syringyl phenols and both cinnamic phenols were most responsive, reaching strong significance (P<0.05). These same four phenols were also most responsive (P<0.05) to the split application of Enersol, but levels of significance for the other seven phenols were in most cases weaker than for the single application, and their sum did not differ significantly from the control (P=0.245). Again, the oxidized acid phenol versions had weaker levels of significance than did the aldehyde and ketone forms. Percentages of increase for individual phenols above the control ranged for the V4 application from 15 to 30% (mean 24%) and for the split application from -1 to 28% (mean 17%).

**Table 4:** Concentrations of individual phenol compounds (mg phenol 100 mg^-1^ plant organic C) in maize roots for the control and the V4 and split applications of the humic product in the upland transect, 2013 and 2014, together with the significance of the humic product main plot effect and the significances for pairwise comparisons of the V4 and split humic product applications with the control.

Phenol	Humic product treatment			Main plot P	Pairwise comparison	
	Control	V4	Split		V4	Split
2013						
Syringaldehyde	1.893	2.368	2.371	0.066	0.043	0.042
Acetosyringone	0.524	0.671	0.646	0.045	0.024	0.041
Syringic acid	0.391	0.490	0.445	0.119	0.051	0.207
Vanillin	1.131	1.474	1.273	0.128	0.056	0.331
Acetovanillone	0.159	0.203	0.173	0.169	0.079	0.495
Vanillic acid	0.165	0.197	0.164	0.217	0.144	0.934
Ferulic acid	1.055	1.313	1.274	0.053	0.028	0.045
p-Coumaric acid	3.082	3.948	3.954	0.045	0.029	0.029
p-OH-benzaldehyde	0.219	0.276	0.269	0.102	0.056	0.078
p-OH-acetophenone	0.029	0.036	0.034	0.159	0.077	0.154
p-OH-benzoic acid	0.043	0.050	0.047	0.273	0.021	0.525
Total (P11)	8.878	10.601	9.970	0.201	0.088	0.245
2014						
Syringaldehyde	2.423	2.346	2.346	0.725	0.504	0.501
Acetosyringone	0.671	0.655	0.654	0.808	0.597	0.575
Syringic acid	0.499	0.494	0.489	0.899	0.812	0.649
Vanillin	1.104	1.139	1.052	0.544	0.657	0.517
Acetovanillone	0.147	0.153	0.144	0.610	0.557	0.698
Vanillic acid	0.145	0.153	0.141	0.342	0.276	0.754
Ferulic acid	1.122	1.109	1.147	0.691	0.772	0.589
p-Coumaric acid	3.939	3.735	3.864	0.402	0.199	0.616
p-OH-benzaldehyde	0.240	0.236	0.239	0.824	0.570	0.905
p-OH-acetophenone	0.032	0.032	0.031	0.546	0.860	0.403
p-OH-benzoic acid	0.041	0.042	0.044	0.299	0.847	0.165
Total (P11)	10.364	10.094	10.151	0.808	0.551	0.636

Color coding represents phenol families.

By contrast, in the 2014 growing season with abundant precipitation, the four most responsive root phenols (syringaldehyde, acetosyringone, coumaric acid, ferulic acid) showed in the upland transect modest numeric decreases for either humic product treatment in seven of the eight cases (**Table 4**). These decreases amounted to no more than 5.2% of the control concentrations for either rate of humic product application. Five of the other seven root phenols showed small numeric increases with the V4 application, while with the split application six of the seven phenols showed minor decreases. For all 11 phenols and their P11 sum, no main plot effects were significant, and the sole pair-wise comparison effects that remotely neared statistical significance were increases in p-hydroxybenzoic acid with the split application (P=0.165) and in p-coumaric acid with the V4 application (P=0.199). Despite the lack of statistical significance, these mostly negligible responses to the humic product stand in contrast to the large positive increases of 2013.

#### Root carbohydrate responses to the humic product

In the droughty 2013 season, main plot treatment effects of humic product application were insignificant (P>0.10) for all five root carbohydrate monomers (**Table 5**). For pair-wise comparisons of the control to each of the humic product treatments, the sole root carbohydrate response to humic product application that neared significance was a decrease in weak-acid glucose (P=0.109) with the split application. All other pair-wise carbohydrate responses were insignificant.

**Table 5:** Concentrations of individual carbohydrate compounds (g kg^-1^) in maize roots for the control and the V4 and split applications of the humic product in the upland transect, 2013 and 2014, together with the significance of the humic product main plot effect and the significances for pairwise comparisons of the V4 and split humic product applications with the control.

Carbohydrate	Humic product treatment			Main plot P	Pairwise comparison	
	Control	V4	Split		V4	Split
2013						
Arabinose	23.2	24.5	22.0	0.785	0.708	0.731
Galactose	10.6	10.6	9.7	0.789	0.973	0.523
Xylose	90.8	93.1	88.8	0.895	0.758	0.894
Weak-acid glucose	93.3	85.8	79.2	0.248	0.357	0.109
Strong-acid glucose	170.6	159.2	172.3	0.246	0.182	0.822
2014						
Arabinose	15.8	18.0	20.0	0.011	0.054	0.004
Galactose	7.6	10.0	10.4	0.014	0.014	0.007
Xylose	86.0	94.5	97.3	0.029	0.010	0.038
Weak-acid glucose	51.1	52.2	61.5	0.032	0.727	0.017
Strong-acid glucose	158.7	156.3	161.9	0.760	0.711	0.626

In 2014, by contrast, main plot treatment effects showed strongly significant (P<0.05) increases with humic product application for all root carbohydrate monomers except strong-acid glucose (P=0.760). For individual humic product treatments, increases in concentrations were strongly significant (P<0.05) with split application for all monomers except strong-acid glucose (P=0.626), and with the V4 application concentration increases were strong for arabinose (P=0.054), galactose (P=0.0145), and xylose (P=0.0101). Numerically, the split application provided for the greatest concentrations for all five monomers, while the unamended control was numerically the least for all monomers except strong-acid glucose. Percent increase for individual carbohydrate monomers ranged with V4 application from -1 to 32% (mean 11%) and with the split application from 2 to 38% (mean 20%).

### Upland transect stover

#### Stover phenol responses to the humic product

In 2012, the droughtiest of the four growing seasons, main plot treatment effects of the humic product in the upland transect were insignificant (P>0.10) for 10 of the 11 phenols and their sum (P11), the only exception nearing significance being an increase in p-coumaric acid (P=0.146) (**Table 6**). Pair-wise LSD comparisons with the unamended control were also clearly insignificant except for, again, an increase in p-coumaric acid (P=0.062) with the V4 application.

**Table 6:** Concentrations of individual phenol compounds (mg phenol 100 mg^-1^ plant organic C) in maize stover for the control and two humic product treatments in the upland transect, 2012 to 2016, together with the significance of the humic product main plot effect and the significances for pairwise comparisons of the control with humic product applications at V4 and either split (2012-2014) or high (2016) rates.

Phenol	Humic product treatment			Main plot P	Pairwise comparison	
	Control	V4	Split/high		V4	Split/high
2012						
Syringaldehyde	1.920	2.072	2.187	0.521	0.519	0.294
Acetosyringone	0.999	1.067	1.053	0.476	0.278	0.364
Syringic acid	0.633	0.671	0.729	0.507	0.644	0.266
Vanillin	1.396	1.441	1.615	0.592	0.728	0.333
Acetovanillone	0.181	0.185	0.184	0.980	0.856	0.882
Vanillic acid	0.218	0.219	0.250	0.466	0.956	0.286
Ferulic acid	1.190	1.195	1.301	0.651	0.977	0.430
p-Coumaric acid	3.113	3.412	3.238	0.146	0.062	0.398
p-OH-benzaldehyde	0.197	0.208	0.221	0.697	0.701	0.415
p-OH-acetophenone	0.020	0.024	0.029	0.544	0.302	0.772
p-OH-benzoic acid	0.064	0.064	0.072	0.322	0.938	0.198
Total (P11)	9.932	10.556	10.031	0.389	0.211	0.828
2013						
Syringaldehyde	1.779	1.602	1.644	0.267	0.132	0.232
Acetosyringone	0.729	0.733	0.718	0.897	0.914	0.745
Syringic acid	0.504	0.498	0.490	0.843	0.792	0.575
Vanillin	1.246	1.233	1.219	0.941	0.871	0.738
Acetovanillone	0.161	0.158	0.161	0.859	0.676	0.940
Vanillic acid	0.159	0.160	0.161	0.943	0.879	0.744
Ferulic acid	1.123	1.180	1.165	0.475	0.256	0.392
p-Coumaric acid	2.814	2.556	2.586	0.222	0.121	0.161
p-OH-benzaldehyde	0.164	0.151	0.151	0.351	0.228	0.212
p-OH-acetophenone	0.026	0.025	0.027	0.307	0.499	0.374
p-OH-benzoic acid	0.032	0.032	0.033	0.644	0.522	0.865
Total (P11)	8.737	8.328	8.354	0.579	0.366	0.395
2014						
Syringaldehyde	1.675	1.517	1.543	0.291	0.154	0.223
Acetosyringone	0.776	0.687	0.738	0.208	0.090	0.421
Syringic acid	0.494	0.445	0.470	0.275	0.123	0.416
Vanillin	1.279	1.102	1.190	0.102	0.040	0.237
Acetovanillone	0.156	0.140	0.150	0.112	0.049	0.503
Vanillic acid	0.170	0.151	0.163	0.076	0.030	0.319
Ferulic acid	1.020	0.850	0.944	0.080	0.031	0.211
p-Coumaric acid	2.515	2.312	2.387	0.535	0.288	0.490
p-OH-benzaldehyde	0.144	0.137	0.137	0.609	0.409	0.398
p-OH-acetophenone	0.025	0.024	0.024	0.879	0.652	0.700
p-OH-benzoic acid	0.035	0.033	0.034	0.646	0.378	0.762
Total (P11)	8.289	7.397	7.780	0.242	0.107	0.321
2016						
Syringaldehyde	0.951	0.945	0.990	0.723	0.887	0.462
Acetosyringone	0.484	0.499	0.504	0.680	0.557	0.420
Syringic acid	0.292	0.294	0.296	0.934	0.883	0.724
Vanillin	0.896	0.929	0.936	0.628	0.493	0.384
Acetovanillone	0.119	0.122	0.122	0.703	0.502	0.480
Vanillic acid	0.110	0.112	0.112	0.893	0.817	0.651
Ferulic acid	0.708	0.726	0.718	0.786	0.506	0.702
p-Coumaric acid	1.596	1.583	1.677	0.626	0.926	0.389
p-OH-benzaldehyde	0.104	0.102	0.106	0.617	0.804	0.474
p-OH-acetophenone	0.015	0.016	0.015	0.956	0.811	0.976
p-OH-benzoic acid	0.027	0.027	0.027	0.768	0.660	0.793
Total (P11)	5.303	5.340	5.504	0.612	0.745	0.349

Color coding represents phenol families.

Despite the lack of statistical significance, numeric trends were consistent in the 2012 upland transect. The split application provided for numerically greater concentrations than did the control for all 11 phenols: the percent increase for each phenol averaged 14%. The V4 application provided for greater concentrations than did the control for 10 of the 11 phenols. For seven of the 11 phenols, the single application was numerically intermediate between the other two treatments, averaging only a 6% increase above the control for each phenol, while the split application averaged a 14% increase. Except for p-coumaric acid, the other three phenols that were most responsive in the roots did not distinguish themselves from the other seven phenols in the 2012 stover.

In the somewhat droughty 2013 upland transect, main plot effects were insignificant (P>0.10) for all 11 phenols and their sum (P11) (**Table 6**). For individual humic product treatments, the only pair-wise LSD comparisons with the control that neared significance (P<0.10) were decreases for syringaldehyde (P=0.132) and p-coumaric acid (0.121) with the V4 application and for p-coumaric acid (P=0.161) with the split application. Compared to the 2012 stover, numeric differences between the control and the two application treatments were subdued: the percent change from the control for each phenol averaged only a 2% decrease for the split application and a 3% decrease for the single application. In five of six cases for the two application treatments, the aldehyde forms of the syringyl, vanillyl, and p-hydroxybenzyl phenols had the strongest level of significance compared to their ketone and acid equivalents.

In the 2014 upland transect, stover concentrations of individual phenols with either humic product treatment were numerically less than those of the corresponding control in all 22 cases (**Table 6**). Main plot treatment effects for the decreases neared or reached significance for vanillic acid (P=0.076), ferulic acid (P=0.080), vanillin (0.102), and acetovanillone (0.112). Pair-wise comparisons were significant with the V4 treatment for decreases in all three vanillic phenols, ferulic acid, and acetosyringone, while the decreases for the other two syringyl phenols and total phenols neared significance to varying degrees (total phenols P= 0.107, syringic acid P=0.123, syringaldehyde P=0.154). For the split application, none of the phenols neared significance. Percent decreases for individual phenols ranged for the V4 treatment from 4 to 17% (mean 9%) and with the split application only from 1 to 8% (mean 5%). The sum of the phenols decreased from the control by 11% with V4 treatment and by 7% with the split application.

In the 2016 upland transect, individual stover phenols and their sum did not respond significantly to either humic product treatment (**Table 6**). Main plot treatment effects and pair-wise comparisons of the control with either humic product treatment did not approach statistical significance. Although phenols levels tended higher with humic product application, all phenol concentrations of the unamended control were within 2% for those of the V4 treatment and about 4% for the split application.

To summarize the four years of stover sampling, upland phenol concentrations responded differently to humic product application across seasons: trending numerically upward in the droughtiest year (2012), but in the somewhat droughty 2013 season the most responsive phenols decreased with humic product application, and in the year with abundant rainfall (2014) several phenols decreased with product application, with no clear trends in the slightly droughty 2016 season. Principal component analysis of upland stover phenols for all four years failed to visually distinguish stover phenols, humic treatments, or years (data not shown).

#### Stover carbohydrate responses to the humic product

The main plot treatment effect was insignificant (P>0.20) for all five stover carbohydrates in each of the four years (**Table S1**). For pair-wise comparisons of individual humic product treatments with the control, the only carbohydrates that neared significance (P<0.10) were decreases with the single V4 application for xylose in 2012 (P=0.116) and for xylose (P=0.133) and weak-acid glucose (P=0.123) in 2014. Numerically, the split application often provided for lower carbohydrate concentrations. Overall, though, stover carbohydrates did not respond strongly or consistently to humic product application.

### Lowland transect roots

#### Root phenol responses to the humic product

Roots were sampled in the lowland transect only in the 2014 season, and then only for the control and split humic product treatment. The pair-wise comparisons for these two treatments were insignificant (P>0.20) for all phenols except for a decrease in ferulic acid with the split application (P=0.060) (**Table S2**). Numeric trends between the control and the split application were mixed.

#### Root carbohydrate responses to the humic product

The pair-wise comparisons for these two treatments were insignificant (P>0.20) for all carbohydrate monomers except for a decrease in xylose with the split application (P=0.060) (**Table S2**). Numeric trends between the control and the split application were mixed, thus failing to follow the significant positive responses of root carbohydrates in the upland transect of this same year.

### Lowland transect stover

#### Stover phenol responses to the humic product

For the 2012 stover, in the lowland transect, the only main plot treatment effect that neared significance and was consistent for both humic product treatments was decreased ferulic acid (P=0.143) (**Table 7**). A near significant main plot treatment effect for p-hydroxy benzaldehyde (P=0.139) was caused solely by an increase (P=0.119) with the V4 application. For individual humic product treatments, the only additional pair-wise comparisons nearing or reaching significance were decreased ferulic acid (P=0.060) and acetosyringone (0.125) with the split application.

**Table 7:** Individual phenol compounds (mg phenol 100 mg^-1^ plant organic C) in maize stover for the control and two humic product treatments in the lowland transect, 2012 to 2016, together with the significance of the humic product main plot effect and the significances for pairwise comparisons of the control with humic product applications at V4 and either split (2012-2014) or high (2016) rates.

Phenol	Humic product treatment			Main plot P	Pairwise comparison	
	Control	V4	Split/high		V4	Split/high
2012						
Syringaldehyde	2.021	2.170	2.042	0.534	0.314	0.886
Acetosyringone	1.160	1.119	1.100	0.273	0.413	0.125
Syringic acid	0.730	0.729	0.725	0.493	0.986	0.302
Vanillin	1.399	1.477	1.418	0.610	0.351	0.766
Acetovanillone	0.189	0.183	0.185	0.777	0.673	0.507
Vanillic acid	0.232	0.228	0.236	0.654	0.777	0.385
Ferulic acid	1.303	1.233	1.204	0.143	0.271	0.060
p-Coumaric acid	3.670	3.719	3.563	0.449	0.782	0.334
p-OH-benzaldehyde	0.200	0.216	0.195	0.139	0.119	0.618
p-OH-acetophenone	0.035	0.034	0.021	0.670	0.960	0.434
p-OH-benzoic acid	0.066	0.068	0.067	0.717	0.527	0.894
Total (P11)	11.004	11.176	10.757	0.377	0.674	0.314
2013						
Syringaldehyde	1.384	1.642	1.223	0.001	0.003	0.018
Acetosyringone	0.671	0.775	0.644	0.067	0.064	0.545
Syringic acid	0.409	0.493	0.374	0.048	0.061	0.336
Vanillin	1.108	1.280	1.042	0.043	0.052	0.352
Acetovanillone	0.142	0.154	0.138	0.141	0.136	0.578
Vanillic acid	0.139	0.159	0.137	0.089	0.065	0.825
Ferulic acid	1.017	1.067	1.011	0.580	0.414	0.921
p-Coumaric acid	2.135	2.593	1.872	0.002	0.003	0.023
p-OH-benzaldehyde	0.128	0.160	0.118	0.007	0.009	0.211
p-OH-acetophenone	0.022	0.024	0.022	0.455	0.285	0.953
p-OH-benzoic acid	0.029	0.033	0.031	0.222	0.104	0.467
Total (P11)	7.184	8.381	6.612	0.010	0.016	0.130
2014						
Syringaldehyde	1.505	1.492	1.577	0.711	0.908	0.528
Acetosyringone	0.720	0.719	0.749	0.647	0.966	0.446
Syringic acid	0.455	0.387	0.481	0.045	0.060	0.416
Vanillin	1.144	1.160	1.178	0.869	0.800	0.612
Acetovanillone	0.146	0.164	0.148	0.057	0.031	0.794
Vanillic acid	0.153	0.154	0.158	0.273	0.561	0.129
Ferulic acid	1.038	1.042	1.027	0.751	0.871	0.588
p-Coumaric acid	2.393	2.573	2.507	0.343	0.164	0.355
p-OH-benzaldehyde	0.137	0.160	0.140	0.032	0.016	0.644
p-OH-acetophenone	0.022	0.033	0.022	0.001	0.001	0.819
p-OH-benzoic acid	0.034	0.027	0.035	0.001	0.007	0.660
Total (P11)	7.747	7.887	8.022	0.748	0.706	0.464
2016						
Syringaldehyde	0.691	0.666	0.674	0.743	0.470	0.612
Acetosyringone	0.346	0.345	0.353	0.920	0.981	0.726
Syringic acid	0.146	0.194	0.198	0.411	0.297	0.232
Vanillin	0.660	0.684	0.683	0.813	0.578	0.606
Acetovanillone	0.088	0.090	0.091	0.901	0.754	0.673
Vanillic acid	0.072	0.074	0.074	0.736	0.523	0.502
Ferulic acid	0.534	0.540	0.544	0.918	0.817	0.692
p-Coumaric acid	1.442	1.353	1.372	0.371	0.197	0.292
p-OH-benzaldehyde	0.086	0.085	0.084	0.899	0.820	0.658
p-OH-acetophenone	0.017	0.017	0.017	0.933	0.971	0.744
p-OH-benzoic acid	0.0158	0.0156	0.0158	0.093	0.753	0.991
Total (P11)	4.099	4.063	4.105	0.982	0.892	0.970

Color coding represents phenol families.

In 2013, when all stover phenol concentrations were uniformly lower than in 2012, the main plot treatment effect approached or reached significance (P<0.10) for seven of the 11 phenols and for total phenols (**Table 7**). These were driven by the V4 application which gave significantly greater concentrations than the control for those seven phenols and total phenols and also neared significance for p-hydroxybenzoic acid (P=0.104) and acetosyringone (P=0.136). In contrast, however, the split application gave numerically lower concentrations than did the control for nine of the 11 phenols, reaching strong significance for syringaldehyde (P=0.018) and p-coumaric acid (P=0.0228). This landscape was particularly wet during early crop growth stages. Our anecdotal observations in other field trials noted crop growth inhibitions following humic product application to overly wet soils, especially at higher application rates. We speculate this same growth inhibition occurred at the higher application rate in this seasonally wet 2013 lowland landscape.

In the 2014 lowland, the single application at V4 gave significantly greater concentrations than did the control for acetovanillone, p-hydroxybenzaldehyde, and p-hydroxyacetophenone, but significantly smaller concentrations for syringic acid and p-hydroxybenzoic acid (**Table 7**). These effects drove the main plot treatment effects for these five phenols, as the split application had little effect on phenol concentrations.

In the 2016 lowland transect, the sole main plot treatment that neared or reached significance was for a minor decrease in p-hydroxybenzoic acid (P=0.093) (**Table 7**). For either humic product treatment, no pair-wise comparison with the control neared or reached significance for any phenol or their sum. To summarize the lowland stover samples across all four years, isolated significant responses to humic product application occurred for individual phenols. The phenol increases with the V4 application in the 2013 season appear consistent with the upland root phenol increases in that somewhat droughty year. Yet they are opposed by the decreased phenol concentrations with the 2013 split application. Overall, the humic product did not have a consistently strong effect on phenol concentrations in lowland stover.

#### Stover carbohydrate responses to the humic product

For the lowland transect, treatment effects of main plot and individual humic product treatments on carbohydrate concentrations were insignificant (P>0.10) for nearly all cases in 2012, 2013 and 2016 (**Table S3**). The only exceptions were a near significant increase (P=0.108) for galactose in 2013 at the main plot level and a significant decrease in 2016 for weak-acid glucose for the main plot and also V4 application. In 2014, both the main plot treatment and the pairwise comparison of the control with the V4 application were significant (P<0.10) to highly significant (P<0.01) for arabinose, galactose, xylose, and weak-acid glucose, while the pairwise comparison with the split application was significant for arabinose, galactose, and weak-acid glucose. The carbohydrate concentration decreased with humic product application in all these cases except for weak-acid glucose with the V4 application. Other than these decreases in 2014, lowland stover carbohydrates did not respond consistently to humic product application, similarly to the upland stover carbohydrates.

### Internodal stalk, sheath, and leaf lignin in the 2013 upland transect

Measurement of internode stalk lignin above the ear leaf through the acetyl bromide analysis found a slight (7%) yet strongly significant (P=0.033) increase for the main plot treatment with humic product application (**Table 8**). This increase was also strongly significant in pair-wise comparisons of the control with separately the single V4 application (P=0.042) and the split application (P=0.016). Acetyl bromide lignin did not change significantly (P>0.10) for the leaf for either humic product treatment, and the control value was intermediate between those of the humic product treatments.

**Table 8:** Concentrations of acetyl bromide-extractable lignin for the maize internode and leaf immediately above the primary ear, and extractable p-coumaric acid and ferulic acid for the same internode, leaf, and also accompanying sheath, sampled from the control and the V4 and split applications of the humic product in the 2013 upland transect at the milk reproductive growth stage, together with the significance (P) of the humic product main plot effect and the significances for pairwise comparisons of the V4 and split humic product applications with the control.

Plant part	Humic product treatment			Main plot P	Pairwise comparison (P)	
	Control	V4	Split		V4	Split
Acetyl bromide-lignin (g kg^-1^)						
Internode	207	220	222	0.033	0.042	0.016
Leaf	137	130	140	0.154	0.134	0.509
p-Coumaric acid (g kg^-1^)						
Internode	41.7	45.2	41.0	0.149	0.108	0.624
Leaf	9.81	8.87	9.72	0.385	0.210	0.869
Sheath	18.2	19.3	18.8	0.439	0.244	0.414
Ferulic acid (g kg^-1^)						
Internode	7.4	6.7	6.3	0.008	0.030	0.003
Leaf	7.29	6.65	6.53	0.015	0.037	0.006
Sheath	12.4	12.4	12.0	0.627	0.876	0.374

Measurement of p-coumaric acid in the internode by the modified Jung and Phillips method (2010) found an increase by 8% (P=0.108) with the single application at V4, but it did not change significantly with the split application (P=0.624). It did not change significantly (P>0.10) from the control with either humic product treatment for either the leaf or sheath. Ferulic acid decreased significantly (P<0.05) from the control with each humic product treatment and the main plot treatment for both the internode and also leaf. For the sheath it did not differ significantly from the control.

## Discussion

The impetus for this study arose from an unexpected field observation. As described previously, a windstorm flattened unamended maize plants across all 16 border rows in the upland portion of this field, irreversibly bending their stalks near the soil surface. The first maize row that remained standing was the first row amended with the humic product (**Figure 1**). Yet in the lowland landscape of this same field, the windstorm bent maize plants much further across the field, including both treated and unamended plots. These observations prompted an exploratory set of biochemical measurements, but with an uncertain outcome: Hence, we sampled maize roots only in 2013 and 2014, keeping the labor supply for this tedious work aligned with a separate root study. Nevertheless, these preliminary biochemical results are novel findings, and they provide guidance for future field studies of humic product effects on crop growth.

An initial finding was a frequent landscape effect on maize phenol and carbohydrate concentrations, within each year and averaged across both treated and untreated treatments. Within each season, all landscape effects are attributable solely to environmental conditions, as the same maize variety was planted across the entire field. Therefore, the frequent landscape effect illustrates the well-known responsiveness of plant biochemistry to environmental signals. In this study, while acknowledging that wind exposure might differ between the upland and lowland transects, we believe the greater phenol concentrations of the stover and roots in the upland transect were more likely due to droughtier conditions. Lignification, that is phenol enrichment, is a known response of plants to drought stress (Enstone et al., 2003; Le Gall et al., 2015), and specifically of maize (Hu et al., 2009; Zhang et al., 2020), especially in specific segments of the roots (Hazman and Kabil, 2022), which is consistent with our results. Increased lignification of root cell walls has been linked with maintained water uptake and transport within the plant during drought conditions (Fan et al., 2006; Moura et al., 2010). In combination with suberization, it might also reduce leakage of water and water vapor from the roots (Cruz et al., 1992), In our study, stover phenols were also most abundant in the droughtiest year, 2012, compared to the other three years, although a varietal difference cannot be discounted in any comparison between years.

Plant lignification can be promoted by other environmental stresses, including low temperature, nutrient deficiency, ozone, and ultraviolet radiation, as reviewed by Moura et al. (2010); Barros et al. (2015) and Kaur et al. (2012). More broadly, abiotic stresses can strongly influence plant production of secondary metabolites, including phenol production (Akula and Ravishankar, 2011). In our study, the field was managed by a private farmer whose yield goals required adequate fertilization of all nutrients. Within the same landscape position, all other variables were similar across the treatment plots, including temperature, nutrient availability, ozone, or light quantity or quality. Therefore, the only difference between the control and other treatments was the humic product application, and any treatment differences in the biochemical traits within the same landscape position are attributable to humic product application.

We hypothesize that the general reduction in maize carbohydrate levels in the upland transect, thereby opposing the phenol trend, reflects a drawdown of carbohydrates to fuel the increased phenol production, as was directly observed by Hatfield et al. (2008) during lignification of maize stems under both greenhouse and field conditions. Similarly, Ertani et al. (2011) noted an hypothesized effect of increased phenol production on carbohydrate metabolism, by citing Muscolo and Sidari (2006). The pathways for diversion of carbohydrates to create phenols are well known. Multiple pathways can occur, but the primary mechanism in plants is the shikimate pathway for transforming carbohydrates into phenylalanine and other aromatic amino acids, followed by the phenylpropanoid pathway for converting phenylalanine into simple phenols, with a key step catalyzed by phenylalanine lyase (Herrmann and Weaver, 1999; Marchiosi et al., 2020).

Building on the landscape effect, humic product application further affected maize biochemical composition. Despite the incomplete root sampling in the lowland transect, results clearly demonstrated stronger maize responses to the humic product in the upland transect than in the lowland. Unexpectedly, the upland responses to the humic product were more significant in two years of root sampling than in four years of stover sampling. During the droughty 2013 season, most root phenols in the upland transect responded positively to humic product application, while with abundant rainfall in 2014 the clear positive response by the upland roots was expressed through their carbohydrates. These humic product effects on roots appear to have amplified the general landscape response of increased lignification during drought stress. The significant increases in the 2013 root phenols with humic product application did not cause noticeable decreases in root carbohydrates. Thus, we speculate that the increased root lignification with humic product application was made possible by greater total carbohydrate production compared to the control. This mechanism could help explain the contrast between our results and the findings of simultaneous increases in both phenols and carbohydrates with humic product application reported by Schiavon et al. (2010); Ertani et al. (2011); Irani et al. (2021); Sakr et al. (2021). We speculate that increased carbohydrate production with humic product application can be partially or fully consumed by diversion to phenol production, depending on plant type and plant need for lignin. We also note differences in assays used among these studies and different pools of carbohydrates and phenols that were reported.

The upland stover phenols did not respond in close association with the upland root phenols. Specifically, some stover phenols showed mild decreases in the 2013 and 2014 upland transects with humic product application, while other phenols showed mixed numeric responses. The 2014 stover phenol decreases were consistent with mild decreases in those 2014 root phenols that were relatively responsive to humic product application (syringaldehyde, acetosyringone, p-coumaric acid, ferulic acid), but the 2013 stover decreases opposed the clear increases in the 2013 root phenols. We are unaware of any further published information that compares biochemical responses of multiple plant parts to humic product application in field conditions.

There are multiple possible explanations for the clearer responses of roots than stover to the humic product. First, roots might intrinsically be the more responsive plant tissue for humic product application to soil, as was largely the practice in our field studies. Nardi et al. (2002) and Kulikova et al. (2016) both reported that addition of humic materials to the roots of growing plants resulted in their preferential accumulation in the roots, compared to much lower proportions that moved further into the shoot and leaves. Such a greater accumulation of the humic product in the roots of our study might have promoted greater changes in local (i.e., root) maize biochemistry than in stover. Second, Hoover et al. (2019) reviewed the biofuels literature to report mixed responses of stover lignin content to drought, in contrast to the frequent reports of root lignification. Our clearer results in roots than in stover could be consistent with these previous reports. Finally, published studies have not investigated thoroughly the relative timings of root lignification vs. stover lignification in response to drought, especially in growing seasons like 2013 when drought stress and excessive soil moisture both occurred, but at different crop growth stages. In one example, as drought stress was continued for white clover (*Trifolium repens* L.), the multiple enzymes involved in its leaf lignification changed in their relative activities (Lee et al., 2007). In our study, stover biochemistry responded to the landscape; therefore, we speculate that the most likely of these potential explanations for greater root response to the humic product would be a concentration of the applied humic product within the roots.

Syringaldehyde, acetosyringone, p-coumaric acid, and ferulic acid were especially responsive to humic product application in multiple years. Especially, their concentrations increased significantly in the 2013 upland roots with humic product application. In addition, of all 11 stover phenols in the 2012 stover, p-coumaric acid provided the only significant increase with humic product application. Conversely, in the 2014 roots, all four phenols decreased numerically (but not statistically) while root carbohydrate abundance increased significantly during the favorable growing conditions. These four phenols are among the five most abundant phenols for both roots and stover. One partial explanation for their responsiveness might be the prominent roles ascribed to syringaldehyde and p-coumaric acid in later stages of lignification. In monocot plants like maize, the syringyl/vanillyl ratio increases as lignification progresses, suggesting greater relative input of syringyl phenols (Stone et al., 1951; Neish, 1960; He and Terashima, 1991). More specifically, late-stage lignification in monocot plants was shown to primarily involve syringyl phenols or their precursor sinapyl alcohol being acetylated through binding with p-coumaric acid (Lu and Ralph, 1999), while ferulic acid may be primarily deposited at earlier stages of lignification (Boerjan et al., 2003).

The role of syringaldehyde in plant growth might be more focused on lignin formation than that of syringic acid, a less responsive phenol to humic product application in our study. Studying aboveground vegetative tissue of maize, Reeves (1987) found that the relative abundance of extractable phenols as syringaldehyde tripled during a growing season from 8.6% to 27.7%, presumably due to its pivotal role in late-season lignification, while that of syringic acid did not increase during the growing season from its initial low level, and in fact it decreased mildly from 7.4% to 6.5%.

In contrast to the phenols, none of the carbohydrate monomers showed special responsiveness to landscape position or the humic product. In part, this might reflect the multiple origins of both glucose pools and their highly variable levels.

These phenol results gained by our conventional cupric oxide extraction for whole plant stover in the 2013 upland transect do not fully agree with trends in acetyl bromide lignin and NaOH-extractable p-coumaric acid and ferulic acid that were analyzed for one internode, sheath, and leaf in that somewhat droughty landscape. As described above, whole stover phenol concentrations (cupric oxide) for the 2013 upland showed no large responses to humic product application, other than significant decreases in p-coumaric acid in both humic product treatments. Numeric trends were mixed, but many of the phenol monomers trended slightly downward. Yet the internode showed increases in acetyl-bromide lignin for both humic product treatments compared to the control and increased p-coumaric acid with the V4 application. These trends are consistent with the increased root lignification described above for humic product application in droughty conditions.

Paradoxically, though, humic product application led to decreased concentrations of NaOH-extractable ferulic acid in both the internode and leaf. Otherwise, the leaf and stalk were unresponsive to humic product application for acetyl bromide and NaOH-extractable p-coumaric acid and ferulic acid. The decrease in ferulic acid cannot be easily reconciled with the cupric oxide results, given differences in both analytical method and in the portion of the maize plant that was sampled.

One possible explanation is the fact that that ferulic acid is subject to esterification *via* cross-coupling reactions with arabinoxylans or other polysaccharides during incorporation into plant cell walls (Harris and Trethewey, 2010). Radical coupling reactions can also occur, resulting in formation of multiple types of bonds, including C-C bonds that cannot always be easily broken by extraction, Meanwhile, p-coumaric acid does not appear to enter into such reactions (Russell et al., 1999). Depending on the extractant and perhaps the stage of lignification, it is possible that increased binding of ferulic acid within the cell wall could lead to its decreased extractability, even with greater potential total accumulation. Separately, analysis of a single internode may well indicate precise insights into growing conditions at the time that internode developed, as opposed to the season-long blend of conditions represented by whole stover analysis through CuO oxidation. Our data do not allow distinction of these possible explanations for divergent results. A thorough integration of the results could be best acquired through frequent plant sampling throughout a growing season for analyses by the acetyl bromide, NaOH extraction, and CuO methods.

The two humic product treatments did not differ consistently from each other in their crop effects across years, landscapes or plant biochemical type. Each humic treatment provided statistically stronger differences from the control than did the other humic treatment in a few but not all cases, and there was no apparent pattern in these cases. We offer no explanation for these random patterns of significance.

The greater effects of the humic product on plant biochemistry in the upland transect than in the lowland transect is consistent with our field observations of 2012, when humic product-treated plants resisted wind damage in the upland transect but not in the lowland transect. Our future studies of maize biochemical responses will determine whether humic product application in droughty conditions causes lignification of primarily the roots and also the stalk base but less so the upper portions of the stover. This hypothesis is consistent with recent field observations on the sturdiness of maize stalk bases with humic product application following harvest. It would also be consistent with previous reports of drought causing maize root lignification but not clearly stover lignification, and with our field observations of the 2012 stalk breakage occurring 10-20 cm above the soil surface.

Our results demonstrate gradational changes in the degrees of maize lignification and carbohydrate loading with humic product application by annual weather pattern and landscape position. They indicate complex interactions between plant biochemical pathways, environmental signals, and the humic product. Such complexity cannot be explained through nutrient-based mechanisms that merely postulate improved availability of soil nutrients. Instead, our results are consistent with the widely held view among researchers of humic products as biostimulants, which promote plant growth through stimulation of cellular-level plant processes, as discussed by Nardi et al. (2002); Mora et al. (2010); Zandonadi et al. (2013); Berbara and Garcia (2014); Calvo et al. (2014), and Olaetxea et al. (2018). This manuscript did not seek to identify the chemically active components of humic products that invoke plant responses. Its results also do not prove that other humic products would cause the same biochemical responses of maize roots and stover. However, we note the vast similarities in agronomic responses of maize grain yield, stover biomass. cob length, and leaf area to both this humic product, created through fine grinding of the source ore (Olk et al., 2021), and to a second humic product created through alkaline extraction (Olk et al., 2022).

The agronomic significance of these results is that increased root lignification and apparent simultaneous drawdown of root carbohydrates provide one plausible mechanism for the observed mitigation of drought stress in maize. Especially in the droughty 2012 season, maize grain yields measured by mechanized combine showed sizable increases with humic product application in the upland transect, while the lowland transect showed more modest increases in grain yield (Olk et al., 2021). In the somewhat droughty 2013 season, the two humic product treatments produced numeric increases in combine grain yield for the upland transect, and one reached statistical significance, while insignificant, numerically mixed trends were found in the lowland transect. The greater upland response to the humic product is consistent with greater drought tolerance induced by the humic product. In the much more favorable 2014 season, by contrast, grain yields for all treatments in the upland transect were considerably higher than under the drought conditions of 2012, showing only subdued responses to humic product application (Olk et al., 2021). Thus, the main plant biochemical response to humic product application in 2014, an increase in upland root carbohydrates, did not bring perceptible agronomic benefit. Separately, we have no clear explanation why the upland root increases in carbohydrate for 2014 was not detected in roots of the one lowland humic treatment in that same year.

**Figure 3:**
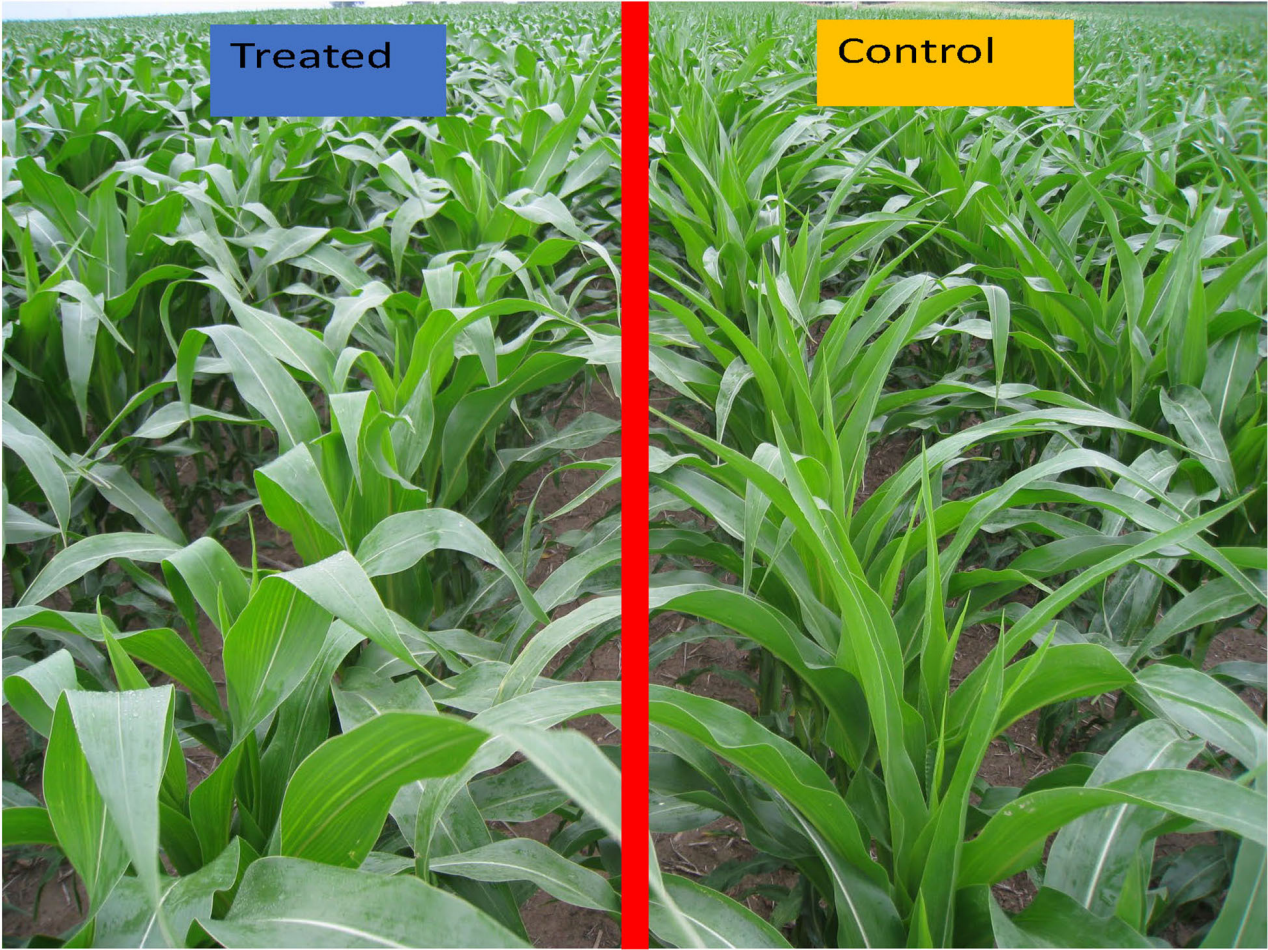
Prevention of wilting in maize at a vegetative growth stage when amended with the humic product (left), compared to the unamended control (right), droughty 2012 season.

The 2014 results lead us to hypothesize that increased carbohydrate production might be the intrinsic plant biochemical response to humic product application in the absence of environmental stresses. In less favorable settings, the increased carbohydrates create options for plant mitigation of environmental stresses, for example increased substrate for root lignification. In addition, increased drought tolerance with humic product application in our study was observed in the droughty 2012 season well before late-season lignification, already by the 8^th^ or 9^th^ leaf growth stage: leaf curling was more pronounced in the unamended control than in an adjacent amended plot (**Figure 3**). Increased production of soluble leaf carbohydrates was reported as an early response of drought-tolerant maize to drought stress (Barnaby et al., 2013). Increased carbohydrate production with humic product application in other settings would provide greater economic returns for crops which are harvested for their carbohydrates. Accordingly, these crops have been identified as among the most responsive crops to humic products, including sugar beet and potato (Khristeva and Manoilova, 1950; Khristeva, 1953), and sugar cane in our unrelated field trials (unpublished data).

## Conclusions

In a four-year field trial in central Iowa, U.S., humic product application to maize during a somewhat droughty year promoted enrichment of root phenolic monomers in a droughty upland transect, especially those phenols associated with late-season lignification. Yet humic product application in the subsequent year with favorable precipitation resulted in only minor effects on those same root phenols, but simultaneously an enrichment of most root carbohydrate monomers. Humic product effects on stover phenol and carbohydrate concentrations were subdued in the upland transect and inconsistent or negligible in a less droughty lowland transect. Such gradational changes in the degrees of maize lignification and carbohydrate loading by annual weather pattern and landscape position indicate complex interactions between plant biochemical pathways, environmental signals, and the humic product. Humic product studies should further investigate the plant processes responding to humic product application in field conditions, and how crop type and environmental stresses alter the accruing economic returns.

## Data availability statement

The original contributions presented in the study are included in the article/**Supplementary Material**. Further inquiries can be directed to the corresponding author.

## Author contributions

DD and DO contributed to the design of the study. DD and DO managed the field experiments and together with RH oversaw the sample analyses. DO, DD, and RH developed the interpretations. DO and DD drafted the manuscript. All authors contributed to the article and approved the submitted version.

## Footnotes

1 Reference to any specific commercial product is for the information of the public and does not constitute endorsement or recommendation by the US government or other sponsors of this report.
