# Supplementary material for: Variable humic product effects on maize structural biochemistry across annual weather patterns and soil types in two Iowa (U.S.A.) production fields
Source: Front Plant Sci. 2023 Jan 12;13:1058141. doi: 10.3389/fpls.2022.1058141 (PMC9878286; doi:10.3389/fpls.2022.1058141)
Supplement: Supplementary file 1 [file DataSheet_1.pdf]

SUPPLEMENTARY MATERIAL

Table S1. Concentrations of individual carbohydrate compounds (g kg^{-1}) in maize stover for the control and two humic product treatments in the upland transect, 2012 to 2016, together with the significance (P) of the humic product main plot effect and the significances for pairwise comparisons of the control with humic product applications at V4 and either split (2012-2014) or high (2016) rates.

Carbohydrate	Humic product treatment			Main plot P	Pairwise comparison	
	Control	V4	Split/high		V4	Split/high
2012						
Arabinose	27.6	28.8	28.5	0.488	0.266	0.397
Galactose	9.5	9.5	9.8	0.928	0.980	0.734
Xylose	139.5	147.9	143.7	0.262	0.116	0.394
Weak-acid glucose	124.2	136.3	123.5	0.688	0.481	0.966
Strong-acid glucose	58.6	77.2	66.3	0.714	0.433	0.741
2013						
Arabinose	22.7	22.5	22.0	0.420	0.896	0.461
Galactose	6.4	5.7	6.0	0.649	0.410	0.530
Xylose	92.9	101.6	90.3	0.205	0.246	0.888
Weak-acid glucose	39.3	45.8	32.5	0.514	0.858	0.970
Strong-acid glucose	7.4	7.3	7.4	0.714	0.952	0.690
2014						
Arabinose	18.6	17.9	17.2	0.403	0.497	0.196
Galactose	6.9	7.0	6.5	0.405	0.813	0.307
Xylose	107.5	100.3	102.6	0.368	0.133	0.286
Weak-acid glucose	48.6	38.1	44.8	0.269	0.123	0.538
Strong-acid glucose	185.3	178.2	185.0	0.870	0.654	0.984
2016						
Arabinose	20.0	19.1	18.9	0.741	0.582	0.476
Galactose	7.9	7.4	7.5	0.853	0.599	0.660
Xylose	89.6	90.2	87.3	0.836	0.904	0.667
Weak-acid glucose	31.4	32.8	31.3	0.804	0.584	0.992
Strong-acid glucose	162.1	161.3	162.0	0.983	0.874	0.993

Table S2. Concentrations of Individual phenol and carbohydrate compounds in maize roots for the control and the split application (Split) of the humic product in the 2014 lowland transect, together with the significance (P) of the humic product main plot effect. Color coding represents phenol families.

Compound	Humic product treatment			Main plot P
	Control	Split		
Phenols (mg phenol 100 mg^{-1} plant organic C)				
Syringaldehyde	2.217	2.226		0.947
Acetosyringone	0.613	0.620		0.855
Syringic acid	0.486	0.480		0.838
Vanillin	1.108	1.092		0.551
Acetovanillone	0.148	0.144		0.399
Vanillic acid	0.158	0.151		0.336
Ferulic acid	1.134	1.090		0.060
p-Coumaric acid	3.679	3.720		0.865
p-OH-benzaldehyde	0.225	0.231		0.624
p-OH-acetophenone	0.032	0.031		0.634
p-OH-benzoic acid	0.042	0.043		0.482
Total (P11)	9.842	9.827		0.976
Carbohydrates (g kg^{-1})				
Arabinose	19.2	18.6		0.548
Galactose	10.9	10.2		0.335
Xylose	123.1	115.6		0.060
Weak-acid glucose	41.2	41.5		0.993
Strong-acid glucose	181.7	198.8		Not determined

Table S3. Concentrations of individual carbohydrate compounds (g kg^{-1}) in maize stover for the control and two humic product treatments in the lowland transect, 2012 to 2016, together with the significance (P) of the humic product main plot effect and the significances for pairwise comparisons of the control with humic product applications at V4 and either split (2012-2014) or high (2016) rates.

Carbohydrate	Humic product treatment			Main plot P	Pairwise comparison	
	Control	V4	Split/high		V4	Split/high
2012						
Arabinose	31.2	29.0	31.2	0.390	0.239	0.973
Galactose	10.4	9.8	9.9	0.569	0.360	0.434
Xylose	167.9	158.2	172.3	0.242	0.248	0.581
Weak-acid glucose	125.0	143.9	146.9	0.313	0.215	0.189
Strong-acid glucose	73.9	58.1	80.4	0.140	0.155	0.530
2013						
Arabinose	24.1	22.2	24.7	0.344	0.293	0.714
Galactose	5.7	5.7	6.9	0.108	0.458	0.317
Xylose	98.7	102.7	99.7	0.700	0.432	0.829
Weak-acid glucose	51.4	51.7	61.2	0.508	0.980	0.321
Strong-acid glucose	6.9	7.0	7.1	0.780	0.606	0.522
2014						
Arabinose	25.0	16.1	21.8	0.0003	0.0001	0.0176
Galactose	9.2	4.3	8.4	<0.0001	<0.0001	0.075
Xylose	135.4	89.7	136.2	<0.0001	<0.0001	0.816
Weak-acid glucose	24.4	40.5	8.0	0.020	0.058	0.094
Strong-acid glucose	144.2	129.4	154.7	0.205	0.280	0.430
2016						
Arabinose	17.2	17.8	18.4	0.472	0.519	0.240
Galactose	6.2	6.5	6.9	0.400	0.544	0.194
Xylose	83.1	86.1	88.3	0.535	0.519	0.285
Weak-acid glucose	41.1	34.7	38.9	0.012	0.004	0.173
Strong-acid glucose	147.5	162.5	156.9	0.351	0.169	0.363
